# The Usefulness and Reliability of Coagrasper for Artery Bleeding during Endoscopic Necrosectomy

**DOI:** 10.3390/medicina59101861

**Published:** 2023-10-19

**Authors:** Yuki Ito, Mitsuru Okuno, Keisuke Iwata, Masahiro Kawade, Yuhei Iwasa, Akihiko Sugiyama, Youichi Nishigaki, Eiichi Tomita

**Affiliations:** Department of Gastroenterology, Gifu Municipal Hospital, Gifu 5008513, Japan; na9yu25@gmail.com (Y.I.); keisukeiwata@nifty.com (K.I.); mabo.lu2239@ezweb.ne.jp (M.K.); festinalenteyu@gmail.com (Y.I.); billsugiyama1025@yahoo.co.jp (A.S.); something@beach.ocn.ne.jp (Y.N.); etomita_jp@yahoo.co.jp (E.T.)

**Keywords:** endoscopic necrosectomy, walled-off necrosis, arterial bleeding, coagrasper, cautery

## Abstract

Although endoscopic necrosectomy (EN) is a less invasive therapy for walled-off necrosis (WON), arterial bleeding can occur during EN. A 60-year-old man with infected WON underwent the EN procedure. During EN, the artery in the WON cavity was injured. As the artery was directly visible, we grasped it using a Coagrasper and coagulated the bleeding point. However, the bleeding was aggravated after coagulation owing to an extension of the vessel damage. The entire vessel was grasped, and complete hemostasis was achieved. The Coagrasper is useful for managing arterial bleeding; however, it should be employed only on the basis of its characteristics and in suitable scenarios.

## 1. Introduction

Endoscopic necrosectomy (EN) for treating walled-off pancreatic necrosis (WON) is less invasive and more life-saving than surgery. However, arterial bleeding during and after EN can occur in 10–20% of cases [1,2,3]. Some useful hemostatic methods, including the use of endoscopic clips and vascular embolization using interventional radiology (IR), have been reported in arterial bleeding therapy during EN [4]. However, these methods have shown some drawbacks as the clips remain within the WON cavity [5] and the IR must temporarily interrupt the EN. Cautery hemostasis using a Coagrasper (Olympus, Tokyo, Japan) has been reported to have a high hemostatic effect that is achieved through directly grasping and coagulating the blood vessels for active gastrointestinal bleeding, as well as during endoscopic submucosal dissection procedures [6,7]. Herein, we report the effectiveness of using the Coagrasper for managing arterial bleeding due to arterial injury during EN for WON treatment, including the precautions regarding its use.

## 2. Case Presentation

A 60-year-old man without previous medical history presented to our hospital with severe abdominal pain. His heart rate was regular, at 69 beats/min, his blood pressure was 120/78 mmHg, and his body temperature was 36.7 °C. The patient was diagnosed with pancreatitis due to a common bile duct stone (CBDS) and underwent endoscopic sphincterotomy (ES) to remove it. However, although the CBDS was completely removed, the pancreatitis was worsened, and an acute kidney injury and respiratory failure were observed. The patient was treated using an artificial respirator and continuous hemodiafiltration in the intensive care unit.

**Figure 1:**
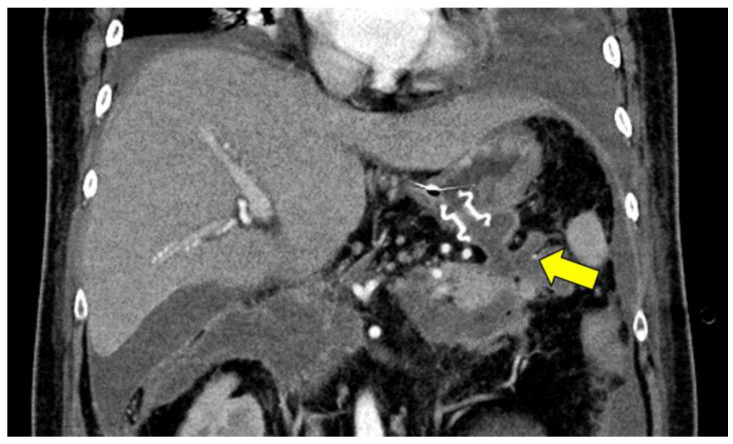
CT after 40 days of admission. WON is observed around the pancreas. The small vessels are observed in the pancreatic tail WON cavity (arrow). LAMS is placed into the pancreatic tail side WON cavity.

The patient’s condition improved with intensive therapy 38 days after undergoing the ES. However, computed tomography revealed a walled-off necrosis (WON) around the pancreas. An infected WON was considered because an elevated C-reactive protein level (8.8 mg/dL) and high fever were observed. Endoscopic ultrasound-guided transgastric drainage for the pancreatic tail WON cavity using a 15 mm lumen-apposing metal stent (LAMS) (Hot Axios; Boston Scientific, Marlborough, MA, USA) was performed (Figure 1), followed by EN, which was performed using five-legged forceps.

**Figure 2:**
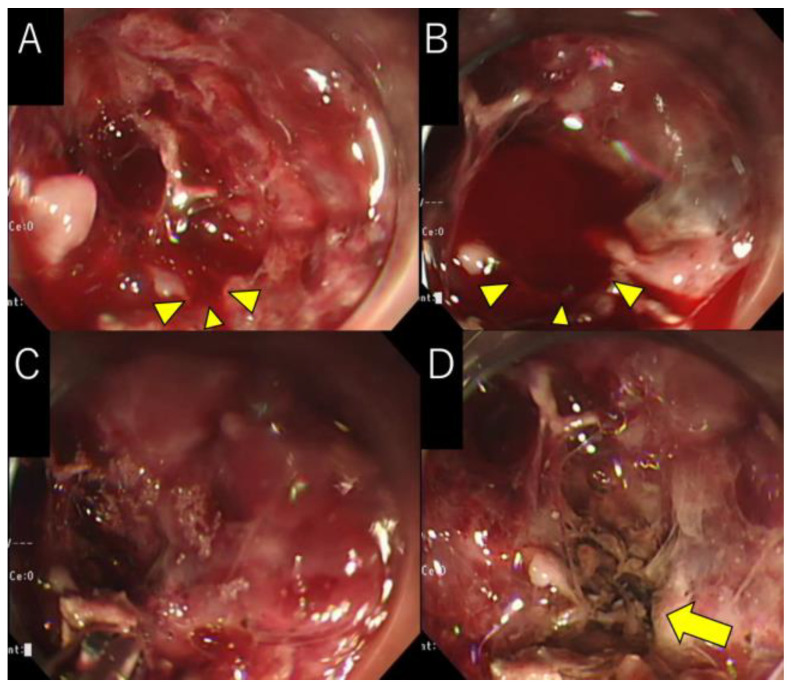
Endoscopic necrosectomy: (**A**) The vessel is damaged during EN, and arterial bleeding (arrowhead) is observed. (**B**) When only the bleeding point is grasped and coagulated using Coagrasper, the blood vessel damage is spread (arrowhead). (**C**) The entire blood vessel is grasped and coagulated using Coagrasper. (**D**) Complete hemostasis is achieved (arrow).

WON is observed around the pancreas. The small vessels are observed in the pancreatic tail WON cavity (arrow). LAMS is placed into the pancreatic tail side WON cavity. During the fourth session, the arterial wall was damaged by forceps, and active bleeding was observed (Figure 2A). Because the artery could be directly observed and was 2–3 mm in diameter, we considered the “Coagrasper” to grasp and coagulate the vessel to achieve hemostasis. Therefore, we grasped and coagulated the bleeding point using the electrosurgical unit ERBE VIO 300D (setting, SOFT COAG mode; effect, 5; max watts, 80; ERBE, Tubingen, Germany); however, the bleeding was aggravated after coagulation owing to the spread of vascular damage. We then grasped the entire vessel and repeatedly coagulated it. Finally, complete hemostasis was achieved (Figure 2C,D, Supplemental Video S1). No bleeding or adverse events were observed 3 days after the vascular cautery. The patient could avoid undergoing vascular embolization with IR and continued the EN procedure. Late adverse events related to the use of the Coagrasper were not evaluated until the completion of the EN procedure.

## 3. Discussion

Only two studies have reported on the use of Coagrasper for arterial bleeding during EN [8,9]. These reports documented successful hemostasis for small-vessel bleeding within the gastric wall or the WON cavity. When most vessels were covered by surrounding tissues, hemostasis was achieved by cauterizing the bleeding point with the surrounding tissues. In this report, we also demonstrated the usefulness of the Coagrasper for obtaining hemostasis in completely denuded vessels in the WON cavity. However, as demonstrated in Figure 2A, inadequate grasping of the vessel can worsen the condition due to the spread of vascular damage. As the artery in our case was not covered by surrounding tissue, grasping the entire vessel was necessary for sufficient cauterization. Therefore, whether the targeted area for grasping should solely encompass the bleeding point or the entire vessel should be ascertained while considering the condition of the vessel (whether it is covered by surrounding tissues or fully exposed) before using the Coagrasper. Moreover, because the Coagrasper has a 5 mm aperture diameter, a blood vessel with a 2–3 mm diameter is considered susceptible to complete grasping and subsequent cauterization. Therefore, when cauterization of an entire vessel is required, the vessel size must also be evaluated when using the Coagrasper.

Holmes et al. reported the treatment of hemorrhages during EN and emphasized that coil embolization under IR is necessary for pseudoaneurysms [4]. Because the Coagrasper can achieve hemostasis by cauterizing and coagulating vessels and/or tissues, attaining hemostasis for a pseudoaneurysm is challenging. It is often difficult to determine whether the bleeding vessel is a pseudoaneurysm during the EN procedure; therefore, confirming the location of the vessel and presence of a pseudoaneurysm before EN using contrast-enhanced computed tomography (CE-CT) is important for determining Coagrasper use and the safety of EN. Hemostasis using coagulation for gastrointestinal bleeding has been reported; however, a risk of delayed rebleeding or perforation persists due to the burning effect [7]. No studies have reported rebleeding associated with coagulation for WON cavity bleeding; however, the reported number of hemostasis procedures using coagulation is small, and late adverse events may not be sufficiently evaluated. Therefore, although the space in the WON cavity is small, it is preferable to cauterize the vessel after lifting and separating the surrounding tissues to avoid coagulation-related adverse events. 

In summary, the Coagrasper is useful for the coagulation of bleeding from a small artery with a diameter of <3 mm. Because attaining hemostasis for a pseudoaneurysm may be challenging and the use of a Coagrasper requires space for lifting the vessel and separating the surrounding tissues, CE-CT evaluation before EN is necessary to assess the situation around the bleeding point during EN. Under these conditions, the Coagrasper could be useful whenever an endoscope can be inserted and can grasp the whole vessel.

## 4. Conclusions

Hemostasis using the Coagrasper is useful for arterial bleeding during EN. However, because the Coagrasper worsens bleeding due to its use in incompatible cases (vessels), it should only be employed after its characteristics and the cases in which its use is compatible have been understood. To evaluate the utility and limitations of the Coagrasper in detail, more studies are needed in the future.

## Data Availability

Not applicable.

## Supplementary Materials

The following supporting information can be downloaded at: https://www.mdpi.com/article/10.3390/medicina59101861/s1, Supplemental Video S1: The usefulness and reliability of the Coagrasper for artery bleeding during endoscopic necrosectomy are shown.
